# Deep learning-based analysis of 12-lead electrocardiograms in school-age children: a proof of concept study

**DOI:** 10.3389/fcvm.2025.1471989

**Published:** 2025-03-05

**Authors:** Shuhei Toba, Yoshihide Mitani, Yusuke Sugitani, Hiroyuki Ohashi, Hirofumi Sawada, Mami Takeoka, Naoki Tsuboya, Kazunobu Ohya, Noriko Yodoya, Takato Yamasaki, Yuki Nakayama, Hisato Ito, Masahiro Hirayama, Motoshi Takao

**Affiliations:** ^1^ Department of Thoracic and Cardiovascular Surgery, Mie University Graduate School of Medicine, Tsu, Mie, Japan; ^2^ Department of Pediatrics, Mie University Graduate School of Medicine, Tsu, Mie, Japan; ^3^ Department of Clinical Engineering, Mie University Hospital, Tsu, Mie, Japan; ^4^ Department of Electrical and Electronic Engineering, Mie University, Tsu, Mie, Japan

**Keywords:** electrocardiograms, school-age children, screening, congenital heart disease, deep learning, arrhythmia detection

## Abstract

**Introduction:**

The diagnostic performance of automated analysis of electrocardiograms for screening children with pediatric heart diseases at risk of sudden cardiac death is unknown. In this study, we aimed to develop and validate a deep learning-based model for automated analysis of ECGs in children.

**Methods:**

Wave data of 12-lead electrocardiograms were transformed into a tensor sizing 2 × 12 × 400 using signal processing methods. A deep learning-based model to classify abnormal electrocardiograms based on age, sex, and the transformed wave data was developed using electrocardiograms performed in patients at the age of 6–18 years during 2003–2006 at a tertiary referral hospital in Japan. Eighty-three percent of the patients were assigned to a training group, and 17% to a test group. The diagnostic performance of the model and a conventional algorithm (ECAPS12C, Nihon Kohden, Japan) for classifying abnormal electrocardiograms were evaluated using the cross-tabulation, McNemar's test, and decision curve analysis.

**Results:**

We included 1,842 ECGs performed in 1,062 patients in this study, and 310 electrocardiograms performed in 177 patients were included in the test group. The specificity of the deep learning-based model for detecting abnormal electrocardiograms was not significantly different from that of the conventional algorithm. For detecting electrocardiograms with ST-T abnormality, complete right bundle branch block, QRS axis abnormality, left ventricular hypertrophy, incomplete right bundle branch block, WPW syndrome, supraventricular tachyarrhythmia, and Brugada-type electrocardiograms, the specificity of the deep learning-based model was higher than that of the conventional algorithm at the same sensitivity.

**Conclusions:**

The present new deep learning-based method of screening for abnormal electrocardiograms in children showed at least a similar diagnostic performance compared to that of a conventional algorithm. Further studies are warranted to develop an automated analysis of electrocardiograms in school-age children.

## 1 Introduction

Twelve-lead electrocardiogram (ECG) is presumed to be useful for detecting children who have a variety of pediatric cardiovascular diseases or are at risk for premature sudden cardiac death in childhood (1). In Japan, ECG has been mandated in all students in the first year of elementary school, junior high school, and senior high school since 1995 (1). In the mass screening of heart diseases in school, children who are prompted by school ECG screening to undergo secondary screening or detailed examination are extracted based on the national guidelines (1). ECG-based screening, in fact, contributes to the early detection of children with long QT syndrome, hypertrophic cardiomyopathy, pulmonary arterial hypertension, and ventricular non-compaction and children who are at high risk of sudden cardiac death (2–11). However, the introduction of ECG-based screening as a nationwide population-level healthcare system remains controversial internationally, mainly because of its high resource utilization (3, 5, 12, 13).

Automated analysis of 12-lead ECG is widely used in adults and its diagnostic performance has been shown to be high with minimal resource utilization (14–18). In addition, recent studies have demonstrated the efficacy of deep learning-based analysis of pediatric ECGs in detecting congenital heart defects, left ventricular dysfunction, and long-QT syndrome (19–22). However, limited data are available in growing children with a variety of target heart diseases and the diagnostic performance of commercially-available automated analyzers remains unknown, in part because of the lack of ECGs annotated based on a guideline for screening children and of the rarity of each target disease (23).

In this study, we aimed to develop a deep learning-based algorithm to interpret ECGs in school-age children and to compare the diagnostic performance of the algorithm with a conventional algorithm implemented in an ECG machine.

## 2 Materials and methods

This study was approved by the Institutional Review Board at Mie University Hospital (H2019-175).

### 2.1 Patients and ECG data

**Figure 1:**
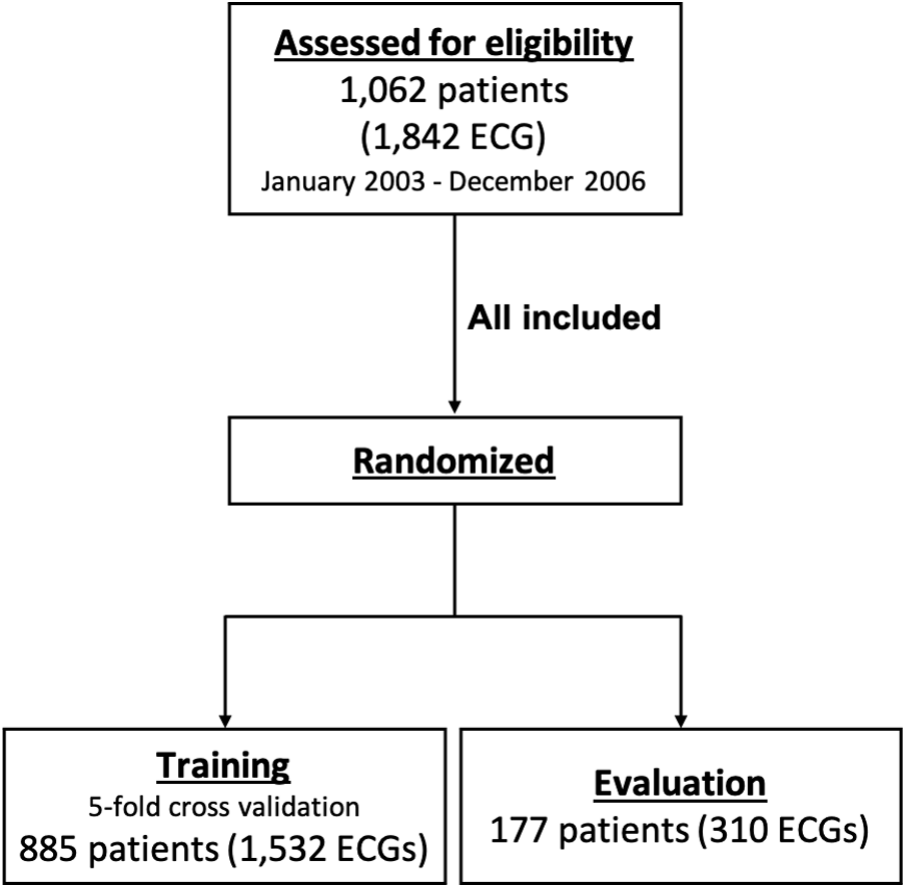
Study population. ECG, electrocardiogram.

We included consecutive patients at 6–18 years of age who underwent a 12-lead ECG at Mie University Hospital, a tertiary referral center from January 2003 to December 2006, during which the same ECG recording system was consistently used. The patients were randomly assigned to the training group (83%), which was used for training the deep learning model, and the test group (17%), which was used for evaluation of the model. The study population is summarized in Figure 1.

In terms of ECG data, standard, simultaneous, digital, 10-s, 12-lead ECGs at rest were originally recorded using ECG-1400 (Nihon Kohden, Japan) in the supine position at a sampling rate of 500 samples per second. Digital wave data of ECG were extracted in the format of medical wave recording format encoding rules (http://www.mfer.org/en/index.htm). In addition to the wave data, findings assigned by an automated algorithm (ECAPS12C, Nihon Kohden, Japan) based on Minnesota coding system (24) and the age and the gender were also extracted.

### 2.2 Interpretation of ECG

As ground truth, ECGs were classified as abnormal when there is a finding included in group A or B in the guideline (1). The findings that were considered abnormal are shown in Supplementary Tables 1–3. All ECGs were interpreted by three board-certified pediatric cardiologists (YM, HS, and HO). When one or more cardiologists considered there was an abnormal finding, the ground-truth diagnosis was determined through discussion by the three cardiologists. During the interpretation process at both stages, the cardiologists had access to the results of automated measurements and diagnoses suggested by the conventional algorithm.

To evaluate the diagnostic performance of the Minnesota coding system-based automated analysis implemented in the ECG machine, Minnesota codes that correspond to the abnormalities included in the group A or B in the guidelines were determined in accordance with guideline (1). The Minnesota codes considered abnormal are shown in Supplementary Table 4. ECGs that had been assigned with these Minnesota codes were considered to be diagnosed as abnormal by the conventional automated algorithm.

### 2.3 Model architecture

As preprocessing of input data, full-length 10-s 12-lead ECG wave data were analyzed using the Pan-Tompkins algorithm, and wave data between the second QRS wave and the last QRS wave were extracted (25). The wave data in each lead were transformed into amplitude and phase using the Fourier transform and filtered using a low pass filter at a threshold of 50 Hz. The amplitude and phase channels were combined based on the Cabrela format and resized to form a three-dimensional tensor sizing 2 × 12 × 400.

**Figure 2:**
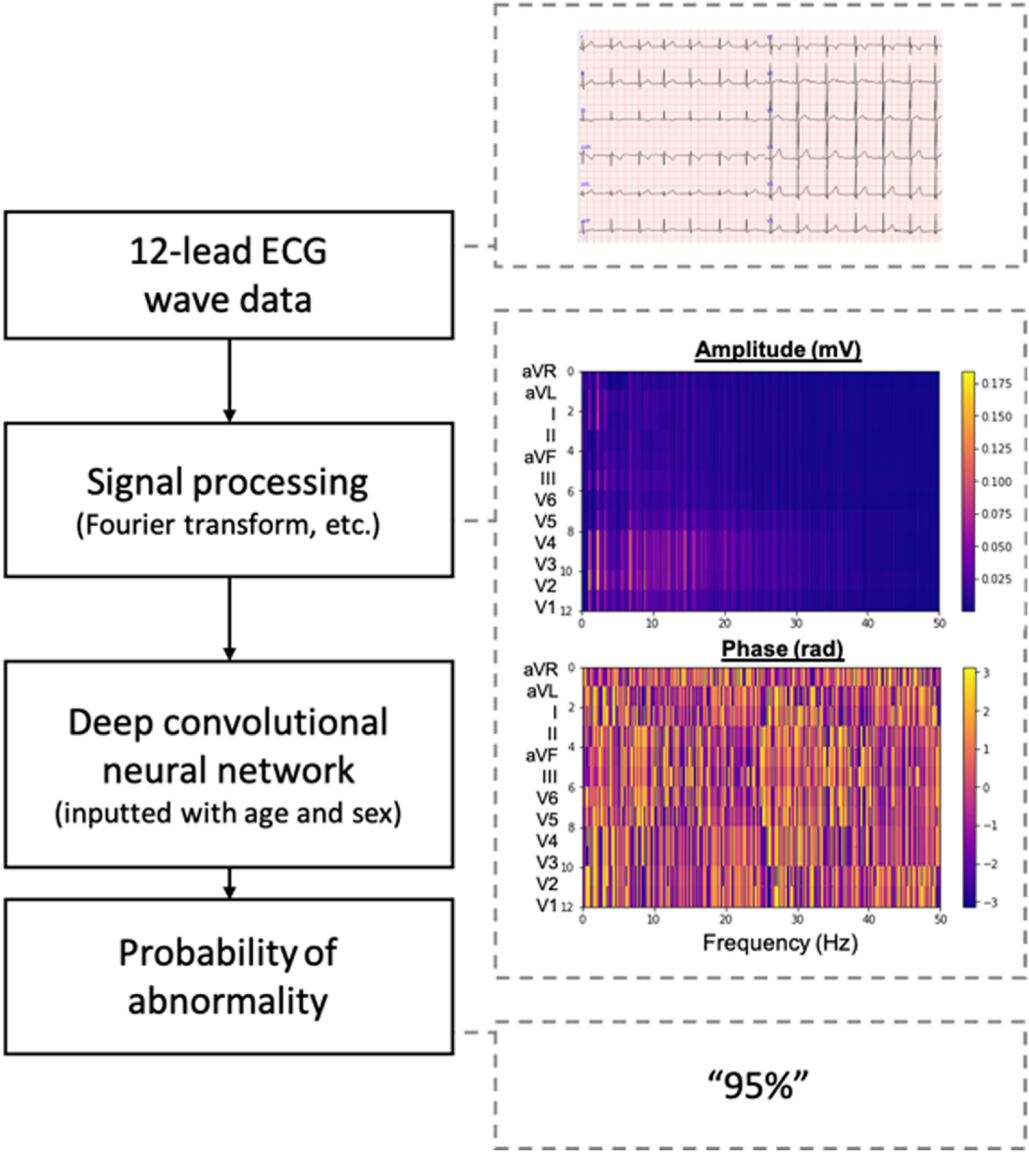
Overview of the deep learning-based model. ECG, electrocardiogram.

**Figure 3:**
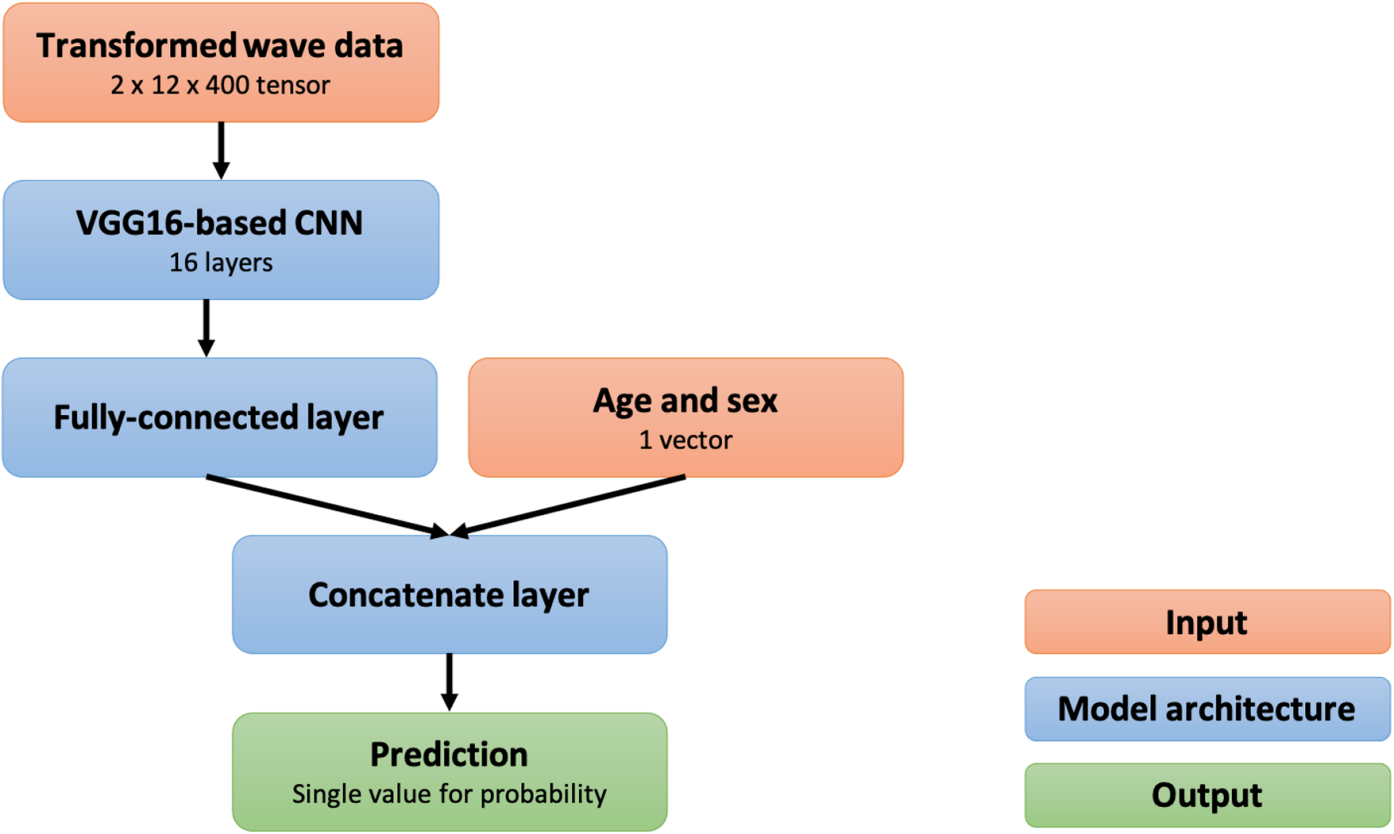
Structural overview of the deep convolutional neural network. CNN, convolutional neural network.

We developed a 21-layer deep convolutional neural network based on the VGG-16 architecture (26). The neural network was designed to have two input layers of the three-dimensional tensor resulting from the preprocessing methods in addition to an input vector for age and sex and to output the probability that an inputted ECG is abnormal. The overview of the preprocessing methods and the deep-learning model is shown in Figure 2, and the architecture of the model is shown in Figure 3.

**Figure 4:**
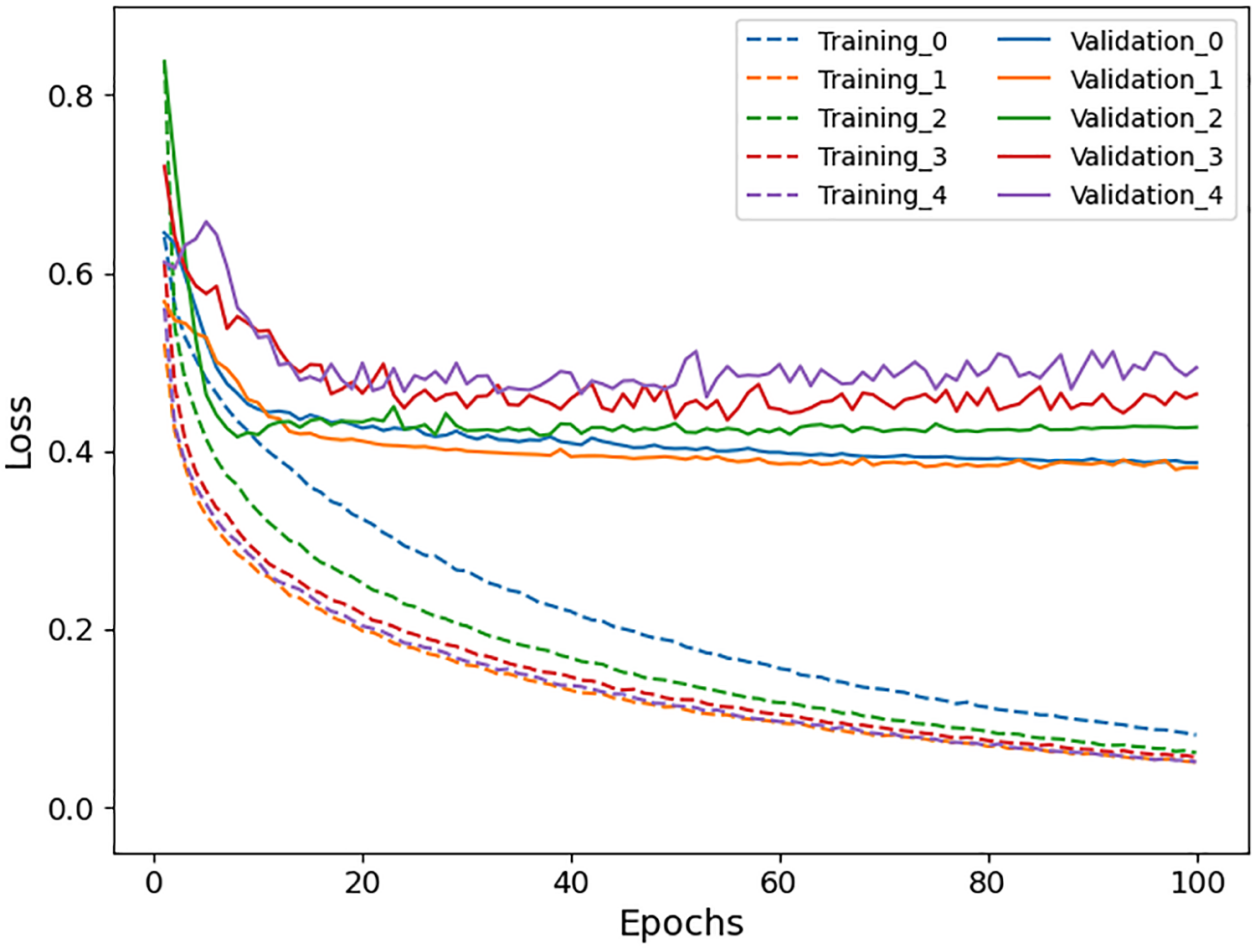
Training curves of the deep learning-based model for 5-fold cross-validation.

The deep learning-based model was trained using data in the training group. The training data were divided into five subgroups, and five models were trained using k-fold cross-validation at k of five (i.e., each model used one of the five training subgroups for validation and the rest for training). After optimization of the parameters using the training data, the following parameters were used for training: batch size, 32; loss function, binary cross entropy; optimizer, Adam; learning ratio, 1.0 × 10^−7^. After training for 100 epochs, models that achieved the lowest loss on validation data were used for performance evaluation. The training curves are shown in Figure 4.

### 2.4 Performance evaluation and statistical methods

The performance of the model to detect abnormal ECGs was evaluated using the area under the receiver operating characteristic curve and the decision curve analysis (27). One threshold for classifying an ECG as abnormal was determined based on Youden's index, and the other threshold was determined so that the sensitivity of the deep learning was as high as the conventional algorithm. At the thresholds determined, accuracy, sensitivity, specificity, positive predictive value, and negative predictive value were calculated and the specificity of the deep learning-based model was compared with that of the conventional algorithm at the threshold for the same sensitivity using the McNemar test (28). Data are presented as median and interquartile range for continuous variables when they are not normally distributed based on the Kolmogorov–Smirnov test.

### 2.5 Visualization

Findings that our model focused on during prediction were visualized by gradient-weighted class activation mapping (grad-CAM) (29). An amplitude channel was produced by using grad-CAM at the 12th convolutional layer of the model and was converted to a heatmap by inverse fast Fourier transform using a phase channel of original ECG data. The heatmap was overlayed on an original ECG wave image to show areas that had influenced the prediction of the model.

### 2.6 Software

Development and evaluation of the deep learning-based model and statistical analyses were performed using python and its libraries including Keras 2.2.4 with TensorFlow backend, scikit-learn, statsmodels, SciPy, and OpenCV.

## 3 Results

### 3.1 Patient characteristics

After excluding three ECGs with corrupted files, a total of 1,842 ECGs performed in 1,062 patients were included in the study. The median age was 11 years (interquartile range, 8–14), and 575 patients (56%) were male. The race of the patients was not recorded, but most (at least 95% or more) were considered to be Asian based on the clinical experience at the center. Of the total ECGs, 1,532 ECGs performed in 885 patients were assigned to the training group, and 310 ECGs performed in the other 177 patients were assigned to the test group. In the test group, 84 ECGs (27%) include one or more abnormal findings as ground truth. Characteristics of ECGs in each group are summarized in Table 1.

**Table 1:** Characteristics of ECG data. The numbers for abnormal ECG findings represent the number of cases in which each finding was present.

Variables	Training group (*n* = 1,532)	Test group (*n* = 310)
Number of patients	885	177
Median age (IQR, years)	11 (8–14)	11 (7–14)
Sex (male, %)	863 (56)	167 (54)
ECG findings (%)		
Normal ECG	1,097 (71)	226 (73)
ST-T abnormality	245 (16)	51 (16)
Complete right bundle branch block	91 (5.9)	17 (5.5)
QRS axis abnormality	51 (3.3)	16 (5.2)
Right ventricular hypertrophy	49 (3.2)	11 (3.5)
Left ventricular hypertrophy	34 (2.2)	5 (1.6)
Left atrial load	31 (2.0)	0
Incomplete right bundle branch block	27 (1.8)	9 (2.9)
Artificial pacemaker	19 (1.2)	0
Q wave abnormality	16 (1.0)	0
Complete left bundle branch block	16 (1.0)	0
Reexamination is required	11 (0.7)	0
Premature supraventricular contraction	10 (0.7)	0
Premature ventricular contraction	9 (0.6)	3 (1.0)
WPW	7 (0.5)	1 (0.3)
Supraventricular tachyarrhythmia	6 (0.4)	8 (2.6)
QT prolongation	5 (0.3)	0
Other rhythm abnormality	4 (0.3)	0
First-degree atrioventricular block	4 (0.3)	0
Second-degree atrioventricular block	3 (0.2)	0
Sinus tachycardia or bradycardia	3 (0.2)	0
Ventricular tachycardia	2 (0.1)	0
Brugada-type electrocardiogram	2 (0.1)	1 (0.3)
Incomplete left bundle branch block	1 (0.1)	0
Intraventricular conduction delay	1 (0.1)	0
Sinus arrest or sinoatrial block	1 (0.1)	0
Third-degree atrioventricular block	0	4 (1.3)

### 3.2 Experts' interpretation

The initial interpretations by the three physicians were the same in 222 ECGs (71%). In the 88 ECGs with disagreement, the findings that were incorrectly not assigned by one or more physicians were: ST-T abnormality (34 ECGs, 39%), QRS axis abnormality (11 ECGs, 13%), left ventricular hypertrophy (5 ECGs, 5.7%), incomplete right bundle branch block (5 ECGs, 5.7%), right ventricular hypertrophy (4 ECGs, 4.5%), third-degree atrioventricular block (3 ECGs, 3.4%), supraventricular tachycardia (3 ECGs, 3.4%), complete right bundle branch block (2 ECGs, 2.3%), and Brugada pattern (1 ECGs, 1.1%). The findings that were incorrectly assigned by one or more physicians were: normal (26 ECGs, 30%), ST-T abnormality (16 ECGs, 18%), incomplete right bundle branch block (8 ECGs, 9.1%), Q wave abnormality (5 ECGs, 5.7%), atrial load (5 ECGs, 5.7%), premature atrial contraction (5 ECGs, 5.7%), prolonged QT (5 ECGs, 5.7%), right ventricular hypertrophy (4 ECGs, 4.5%), QRS axis abnormality (2 ECGs, 2.3%), left ventricular hypertrophy (2 ECGs, 2.3%), Brugada pattern (1 ECG, 1.1%), complete right bundle branch block (1 ECG, 1.1%), 1st-degree atrioventricular block (1 ECG, 1.1%), and other rhythm abnormality (1 ECG, 1.1%).

### 3.3 Performance of the conventional algorithm

The conventional algorithm classified 208 ECGs (67%) as abnormal in the test group. The accuracy, sensitivity, specificity, positive predictive value, and negative predictive value are 0.57 [95% confidence interval (CI), 0.52–0.63], 0.95 (95%CI, 0.93–0.97), 0.43 (95%CI, 0.38–0.49), 0.38 (95%CI, 0.33–0.43), and 0.96 (95%CI, 0.94–0.98), respectively. The accuracy of the conventional algorithm was similar between the ECGs with initial agreement among physicians and those with initial disagreement (0.57 vs. 0.58).

### 3.4 Performance of the deep learning model

**Figure 5:**
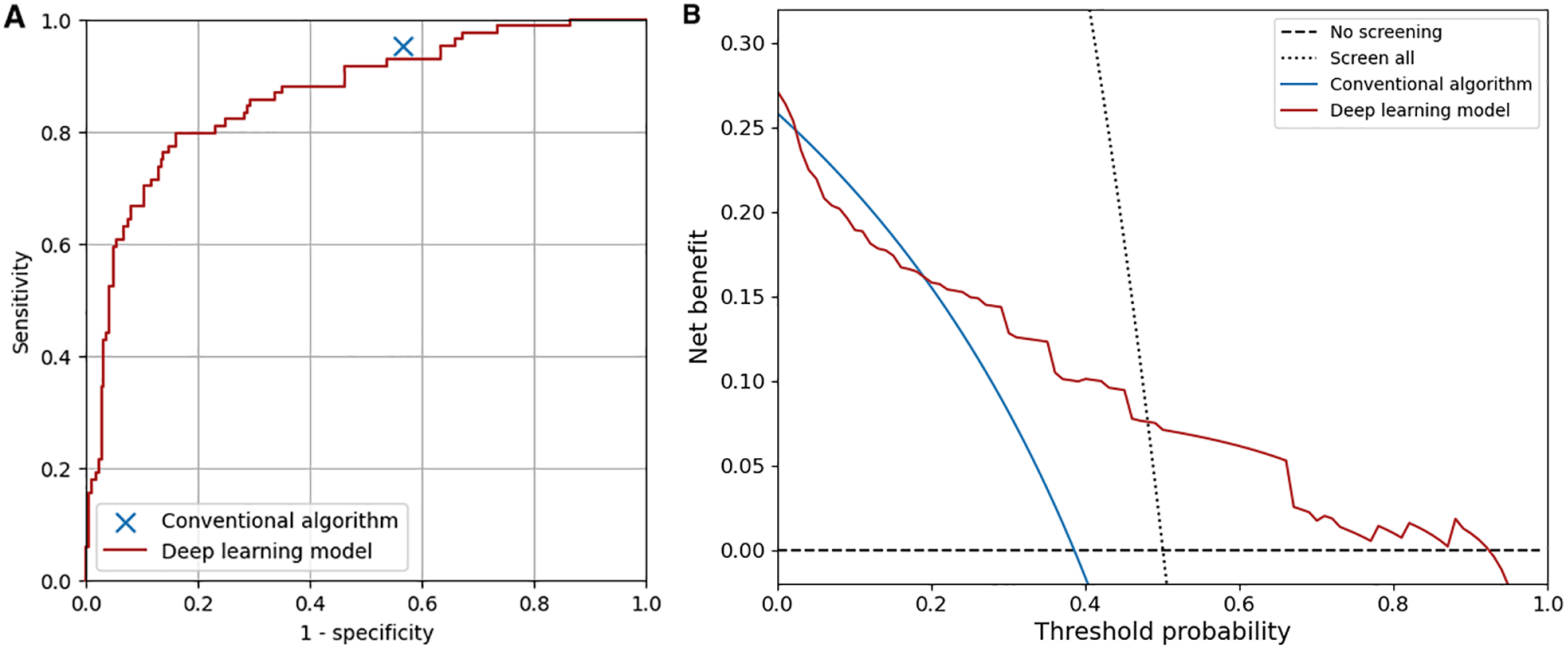
Overall diagnostic performance of the conventional algorithm and the deep learning-based model. **(A)** Receiver operating characteristic curve of the deep learning-based model. The area under the curve was 0.87. **(B)** Decision curves of the conventional algorithm and the deep learning-based model. The test harm was assumed to be 0.

The receiver operating characteristic curve and the decision curve of our model for detecting overall abnormality are shown in Figure 5. The net benefit of the deep learning-based model was higher than that of the conventional algorithm at the threshold probability of 0.04 or lower and 0.19 or higher. At maximum Youden's J index, our model classified 102 ECGs (33%) as abnormal, and the accuracy, sensitivity, specificity, positive predictive value, and negative predictive value were 0.83 (95%CI, 0.79–0.87), 0.79 (95%CI, 0.75–0.84), 0.84 (95%CI, 0.80–0.88), 0.65 (95%CI, 0.60–0.70), and 0.91 (95%CI, 0.88–0.94), respectively. To achieve the same sensitivity (95%) as the conventional algorithm, the threshold for the output of the model was set to 0.022, and the accuracy, sensitivity, specificity, positive predictive value, and negative predictive value were 0.52 (95%CI, 0.47–0.58), 0.95 (95%CI, 0.93–0.97), 0.37 (95%CI, 0.32–0.42), 0.36 (95%CI, 0.31–0.41), and 0.95 (95%CI, 0.93–0.97), respectively. The specificity of the model was not significantly different from that of the conventional algorithm (*P* = .58, McNemar's test). The accuracy of the model was similar between the ECGs with initial agreement among physicians and those with initial disagreement (0.52 vs. 0.52).

### 3.5 Performance for each abnormality

**Figure 6:**
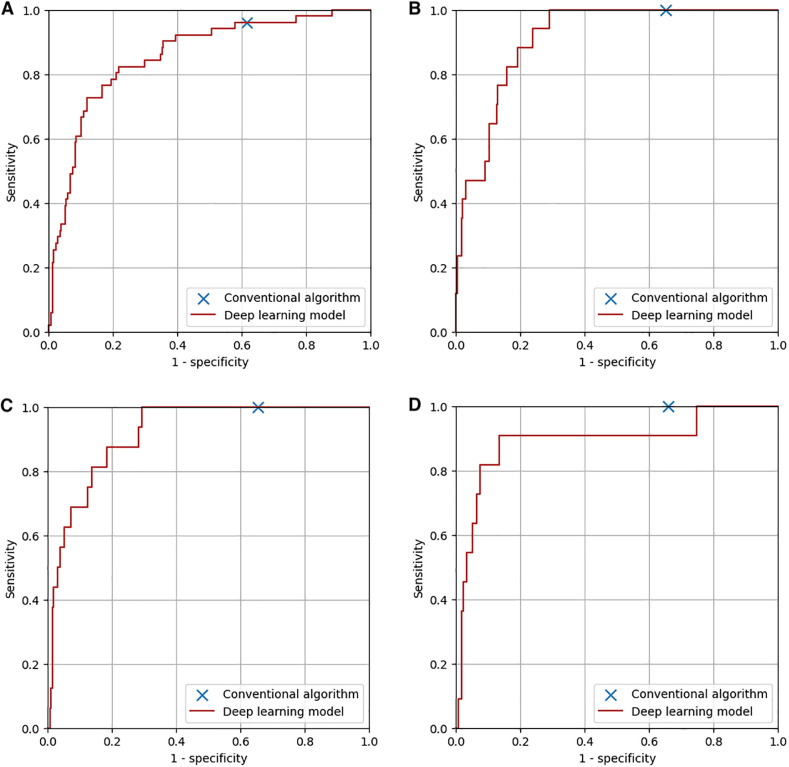
Diagnostic performance of the conventional algorithm and the deep learning-based model for four major abnormalities. **(A)** ST-T abnormality. The area under the curve of the deep learning-based model was 0.86. **(B)** Complete right bundle branch block. The area under the curve of the deep learning-based model was 0.91. **(C)** QRS axis abnormality. The area under the curve of the deep learning-based model was 0.92. **(D)** Right ventricular hypertrophy. The area under the curve of the deep learning-based model was 0.89.

The diagnostic performance of the conventional algorithm and deep learning-based model for abnormalities for which both training and test groups contain one or more ECGs with such abnormality at the same sensitivity are shown in Table 2. At the same sensitivity, the specificity of the deep learning-based model was significantly higher for ECGs with ST-T abnormality, complete right bundle branch block, QRS axis abnormality, left ventricular hypertrophy, incomplete right bundle branch block, WPW syndrome, supraventricular tachyarrhythmia, and Brugada-type electrocardiograms, but not significantly different for ECGs with right ventricular hypertrophy and premature ventricular contraction. The receiver operating characteristic curves of the deep learning-based model for four major abnormalities (ST-T abnormality, complete right bundle branch block, QRS axis abnormality, and right ventricular hypertrophy) are shown in Figure 6.

**Table 2:** Diagnostic performance for electrocardiograms with each abnormality. Values are shown with 95% confidence intervals. *P* values were calculated using McNemar's test.

Findings	Conventional algorithm					Deep learning-based model at the same sensitivity					*P* value
	Accuracy	Sensitivity	Specificity	Positive predictive value	Negative predictive value	Accuracy	Sensitivity	Specificity	Positive predictive value	Negative predictive value	
ST-T abnormality	0.48 (0.42–0.54)	0.96 (0.94–0.98)	0.38 (0.33–0.43)	0.24 (0.20–0.29)	0.98 (0.96–1.0)	0.59 (0.54–0.65)	0.96 (0.94–0.98)	0.42 (0.37–0.48)	0.25 (0.20–0.30)	0.98 (0.96–1.0)	0.021
Complete right bundle branch block	0.38 (0.33–0.43)	1.0 (1.0–1.0)	0.35 (0.30–0.40)	0.082 (0.051–0.11)	1.0 (1.0–1.0)	0.82 (0.78–0.86)	1.0 (1.0–1.0)	0.71 (0.66–0.76)	0.17 (0.13–0.21)	1.0 (1.0–1.0)	<0.001
QRS axis abnormality	0.38 (0.33–0.43)	1.0 (1.0–1.0)	0.35 (0.30–0.40)	0.077 (0.047–0.11)	1.0 (1.0–1.0)	0.82 (0.78–0.86)	1.0 (1.0–1.0)	0.71 (0.66–0.76)	0.16 (0.12–0.20)	1.0 (1.0–1.0)	<0.001
Right ventricular hypertrophy	0.36 (0.31–0.41)	1.0 (1.0–1.0)	0.34 (0.29–0.39)	0.053 (0.028–0.078)	1.0 (1.0–1.0)	0.50 (0.44–0.56)	1.0 (1.0–1.0)	0.25 (0.20–0.30)	0.047 (0.023–0.071)	1.0 (1.0–1.0)	0.93
Left ventricular hypertrophy	0.35 (0.29–0.40)	1.0 (1.0–1.0)	0.33 (0.28–0.39)	0.024 (0.007–0.041)	1.0 (1.0–1.0)	0.71 (0.66–0.76)	1.0 (1.0–1.0)	0.51 (0.46–0.57)	0.033 (0.013–0.053)	1.0 (1.0–1.0)	<0.001
Incomplete right bundle branch block	0.35 (0.30–0.40)	0.89 (0.85–0.92)	0.34 (0.28–0.39)	0.039 (0.017–0.060)	0.99 (0.98–1.0)	0.84 (0.81–0.89)	0.89 (0.85–0.92)	0.78 (0.73–0.83)	0.11 (0.07–0.14)	1.0 (0.99–1.0)	<0.001
Premature ventricular contraction	0.34 (0.29–0.39)	1.0 (1.0–1.0)	0.33 (0.28–0.38)	0.014 (0.001–0.028)	1.0 (1.0–1.0)	0.52 (0.47–0.58)	1.0 (1.0–1.0)	0.28 (0.23–0.33)	0.014 (0.001–0.026)	1.0 (1.0–1.0)	0.58
WPW	0.33 (0.28–0.38)	1.0 (1.0–1.0)	0.33 (0.28–0.38)	0.0,048 (0.0–0.013)	1.0 (1.0–1.0)	0.82 (0.78–0.86)	1.0 (1.0–1.0)	0.85 (0.81–0.89)	0.021 (0.005–0.037)	1.0 (1.0–1.0)	<0.001
Supraventricular tachyarrhythmia	0.35 (0.30–0.41)	1.0 (1.0–1.0)	0.33 (0.29–0.39)	0.039 (0.017–0.060)	1.0 (1.0–1.0)	0.83 (0.79–0.87)	1.0 (1.0–1.0)	0.73 (0.68–0.77)	0.088 (0.056–0.12)	1.0 (1.0–1.0)	<0.001
Brugada-type electrocardiogram	0.33 (0.28–0.38)	1.0 (1.0–1.0)	0.33 (0.28–0.38)	0.005 (0.0–0.012)	1.0 (1.0–1.0)	0.83 (0.79–0.87)	1.0 (1.0–1.0)	0.70 (0.65–0.75)	0.011 (0.0–0.022)	1.0 (1.0–1.0)	<0.001

### 3.6 Visualization

**Figure 7:**
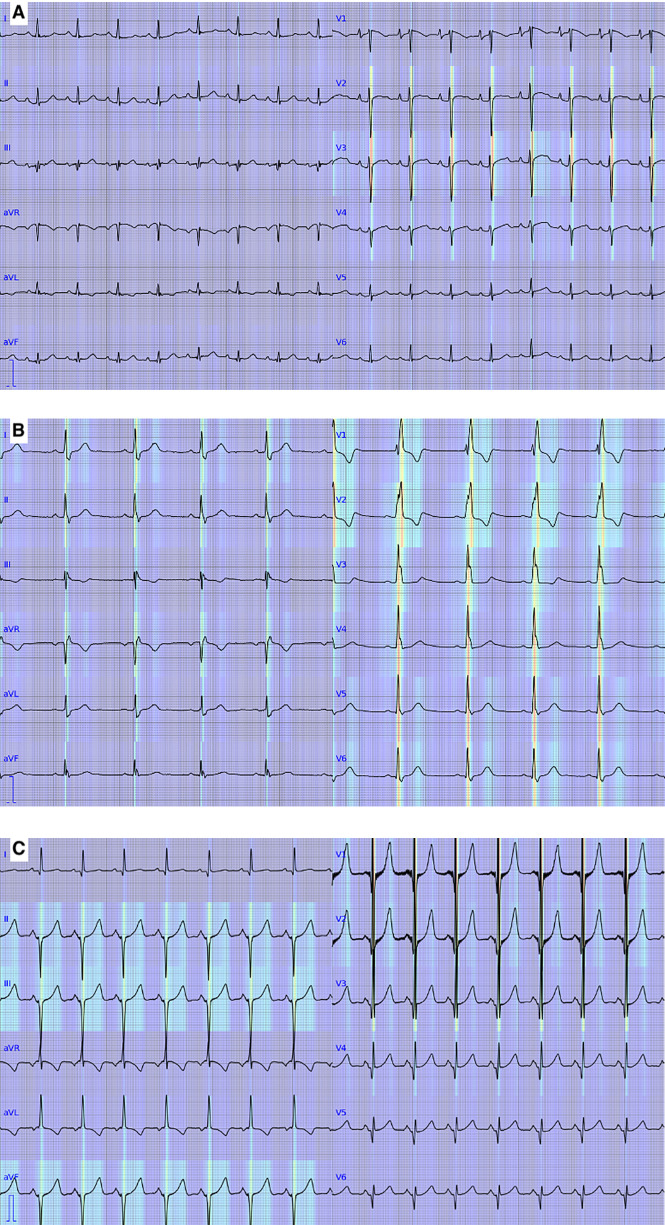
Visualization of the deep learning-based model. Red and yellow represent areas where the model focused on to output predictions. **(A)** An electrocardiogram with ST-T abnormality. **(B)** An electrocardiogram with complete right bundle branch block. **(C)** An electrocardiogram with QRS axis abnormality, right ventricular hypertrophy, and ST-T abnormality.

Interpretation for ECGs with ST-T abnormality, with complete right bundle branch block, and with QRS axis abnormality, right ventricular hypertrophy, and ST-T abnormality by our model was visualized and shown in Figure 7.

## 4 Discussion

In this study, we developed and validated a new deep learning-based algorithm to detect the guideline-defined abnormalities in ECGs in school-age children. To our knowledge, this is the first application of deep learning to interpreting the 12-lead ECG in children with a variety of target heart diseases in accordance with the nationwide population-level screening guideline, and we showed that the deep learning-based analysis could detect abnormalities in ECGs based on wave data, age, and sex, at the diagnostic performance at least similar to the conventional algorithm.

Automated analysis of 12-lead ECG has been studied since 1950s (15, 30). In previous studies, automated analysis of ECG was performed using the rule-based approach, in which ECG waveforms were recognized and interpreted based on descriptive rules. The rule-based automated analysis of ECG has been reported to have high diagnostic performance in adults (14, 16, 17). However, its application to ECGs in children has been limited, and to our knowledge, the diagnostic performance of automated algorithms for ECGs in children with a variety of target heart diseases has not been reported. In this study, we aimed to develop a method of automated analysis of ECG in children without defining a detailed description of ECG waveforms using a deep learning-based method.

Although a standard protocol for the interpretation of ECGs for screening in school-age children has been established in Japan, the percentage of students who were regarded as having abnormal ECG varied among districts in Japan (1). In this study, disagreement among the three certified pediatric cardiologists occurred in 29% of the ECGs for evaluation. Considering the relatively high inconsistency rate even among experienced physicians, accurate and consistent analysis of ECGs will help improve the efficacy of screening of ECGs in school-age children.

Deep learning has recently been developed initially in the field of automated image recognition. One of the advantages of deep learning is that deep learning models extract features that are necessary to predict outputs not by descriptive definition of such features but through the learning process in which a model is trained using a large dataset of inputs and outputs. Because of this non-descriptive learning process, it can recognize and interpret images without descriptive definitions of waveforms and abnormalities.

Recent studies have shown superior performance of deep learning-based methods to analyze single-lead ECGs as well as 12-lead ECGs (19–22, 31–34). In some previous studies, deep learning-based methods were applied by inputting 12-lead ECG data as a 2-dimensional array to convolutional neural networks (19, 21, 22, 34–37). However, a typical convolutional neural network does not recognize sequential relationships among time-dependent wave data, especially at distant time points. To handle ECG wave data as time-sequence data, some previous studies used recurrent neural networks, including long short-term memory network for deep learning model (38, 39), time-frequency spectrogram (40, 41), or transformer (20). In the present study, we focused on periodicity in addition to the time sequence of ECG waveforms and developed a model that used conventional signal processing methods, such as the Fourier transform and the Pan-Tompkins algorithm, and deep convolutional networks. The model used in the present study has four theoretical advantages compared with the models used in the previous deep leaning-based ECG studies (34–42). First, the input of the present model is wave data, which is consistent among different recording systems and to which various signal-processing methods, such as low pass filter, can be applied. Second, because of the structure of the input tensor, which was created based on Cabrela format, and the convolutional layers used in the deep learning model, the geometrical relationships among 12 leads of ECG can be recognized in the model. Third, because the input data we used was not time-dependent voltage data nor time-frequency spectrogram but 3-dimensionally concatenated spectrums in which one dimension is for leads, one dimension is for frequency, and the other dimension is for amplitude and phase as results of Fourier transform, we could reduce the size of the input tensor to 2 × 12 × 400. The smaller size of the input tensor compared to the previous studies may have contributed to the performance of the model despite the relatively small number of included patients (35–38, 42). This can be a potential advantage for screening abnormal ECGs in children, who are associated with a variety of rare cardiac diseases. Forth, the present model is a multi-modal deep-learning model that includes age and sex as well as ECG data as its inputs. This may be beneficial to analyze ECGs in growing children of different ages and genders.

In the previous studies, the diagnostic performance of deep learning-based analysis of ECGs in adults was shown to be similar to or superior to that of conventional automated analysis (43, 44). However, the diagnostic performance of deep learning-based or conventional automated analysis for ECGs in children with a variety of target heart diseases by using a screening guideline has not been reported. In this study, we first evaluated the diagnostic performance of a conventional algorithm implemented in an ECG recorder (ECG-1400; Nihon Kohden, Japan) and then compared it with that of deep learning-based analysis. As a result, the conventional algorithm was shown to be sensitive to the abnormalities in children but less specific (sensitivity, 0.95; specificity, 0.43), and the deep learning-based model had similar overall diagnostic performance to that of the conventional algorithm and was more specific for ECGs with some abnormalities, such as ST-T abnormality, complete right bundle branch block, QRS axis abnormality, left ventricular hypertrophy, incomplete right bundle branch block, WPW syndrome, supraventricular tachyarrhythmia, and Brugada-type electrocardiograms. As shown in Figure 5B, the net benefit of the deep learning-based model was higher than that of the conventional algorithm when at the lower and higher ends of the threshold probability. The present convolutional neural network, which recognizes the relationships among waves in adjacent leads in the Cabrela format, may have contributed to the performance of the model. Considering that the performance of the deep learning-based model can be improved by including more data for training, our deep learning-based model can classify ECGs in children at least as accurately as a conventional automated algorithm does.

One of the disadvantages of a deep learning-based model for clinical purposes is that the recognition and interpretation of a model are not well explained, and it is often considered a “black box.” In this study, we showed areas of ECG wave on which our deep learning-based model focused on to output prediction, as shown in Figure 7. The areas were generally consistent with the abnormalities in the abnormal ECGs except for the one with ST-T abnormality. The inconsistency in the ECG with ST-T abnormality may be caused by the frequent association of ST-T abnormality with the other abnormalities in the training data. This visualization technique may help clinicians review the model's predictions.

Several limitations of this study should be acknowledged. First, because some of the ECG findings were rare, several abnormalities in the guidelines were rarely or not included in the training or test dataset. Second, because the output of our deep learning-based model was for overall abnormality (i.e., whether an ECG includes any abnormal findings), the evaluation of our model for each abnormality may not represent the exact performance of the deep learning-based model for each abnormality. Third, the data recorded in the previous period were used in this study due to the limited resources for the meticulous annotation process. The conventional algorithm implemented in the ECG recorder has remained largely the same, with only minor updates. These updates may affect the performance of the current rule-based algorithm. In addition, Minnesota codes were revised in 2010, but this didn't affect the results of the study. These limitations will be solved by including more cases and by developing specific models for each abnormality using a large amount of data in future studies.

In conclusion, we developed a new deep learning-based method to detect abnormalities defined by a national ECG screening guideline in 12-lead ECGs in children, and its diagnostic performance was at least similar to that of a conventional automated algorithm. Further studies are warranted to improve the diagnostic performance of the model for automated analysis of ECGs in the setting of screening for apparently healthy school children.

## Data Availability

The participants of this study did not give written consent for their data to be shared publicly, so due to the sensitive nature of the research supporting data is not available. Requests to access these datasets should be directed to Shuhei Toba, toba.shuhei@gmail.com.

## References

[1] Sumitomo N Baba R Doi S Higaki T Horigome H Ichida F Guidelines for heart disease screening in schools (JCS 2016/JSPCCS 2016)—digest version. Circ J. (2018) 82(9):2385–444. 10.1253/circj.CJ-66-0153 30101812

[2] Sawada H Mitani Y Nakayama T Fukushima H Kogaki S Igarashi T Detection of pediatric pulmonary arterial hypertension by school electrocardiography mass screening. Am J Respir Crit Care Med. (2019) 199(11):1397–406. 10.1164/rccm.201802-0375OC 30428270

[3] Anderson BR McElligott S Polsky D Vetter VL. Electrocardiographic screening for hypertrophic cardiomyopathy and long QT syndrome: the drivers of cost-effectiveness for the prevention of sudden cardiac death. Pediatr Cardiol. (2014) 35(2):323–31. 10.1007/s00246-013-0779-0 24005901

[4] Hirono K Miyao N Yoshinaga M Nishihara E Yasuda K Tateno S A significance of school screening electrocardiogram in the patients with ventricular noncompaction. Heart Vessels. (2020) 35(7):985–95. 10.1007/s00380-020-01571-7 32161993

[5] Wheeler MT Heidenreich PA Froelicher VF Hlatky MA Ashley EA. Cost-effectiveness of preparticipation screening for prevention of sudden cardiac death in young athletes. Ann Intern Med. (2010) 152(5):276. 10.7326/0003-4819-152-5-201003020-00005 20194233 PMC2873148

[6] Williams EA Pelto HF Toresdahl BG Prutkin JM Owens DS Salerno JC Performance of the American Heart Association (AHA) 14-point evaluation vs. electrocardiography for the cardiovascular screening of high school athletes: a prospective study. J Am Heart Assoc. (2019) 8(14):e012235. 10.1161/JAHA.119.012235 31286819 PMC6662133

[7] Yoshinaga M Kucho Y Nishibatake M Ogata H Nomura Y. Probability of diagnosing long QT syndrome in children and adolescents according to the criteria of the HRS/EHRA/APHRS expert consensus statement. Eur Heart J. (2016) 37(31):2490–7. 10.1093/eurheartj/ehw072 27026747

[8] Fukuyama M Horie M Aoki H Ozawa J Kato K Sawayama Y School-based routine screenings of electrocardiograms for the diagnosis of long QT syndrome. EP Europace. (2022) 24(9):1496–503. 10.1093/europace/euab320 35060598

[9] Imamura T Sumitomo N Muraji S Yasuda K Nishihara E Iwamoto M Impact of the T-wave characteristics on distinguishing arrhythmogenic right ventricular cardiomyopathy from healthy children. Int J Cardiol. (2021) 323:168–74. 10.1016/j.ijcard.2020.08.088 32877757

[10] Muraji S Sumitomo N Imamura T Yasuda K Nishihara E Iwamoto M Diagnostic value of P-waves in children with idiopathic restrictive cardiomyopathy. Heart Vessels. (2021) 36(8):1141–50. 10.1007/s00380-021-01784-4 33496817

[11] Yoshinaga M Horigome H Ayusawa M Yasuda K Kogaki S Doi S Electrocardiographic diagnosis of hypertrophic cardiomyopathy in the pre- and post-diagnostic phases in children and adolescents. Circ J. (2021) 86(1):118–27. 10.1253/circj.CJ-21-0376 34615813

[12] Fuller CM. Cost effectiveness analysis of screening of high school athletes for risk of sudden cardiac death. Med Sci Sports Exerc. (2000) 32(5):887–90. 10.1097/00005768-200005000-00002 10795776

[13] Leslie LK Cohen JT Newburger JW Alexander ME Wong JB Sherwin ED Costs and benefits of targeted screening for causes of sudden cardiac death in children and adolescents. Circulation. (2012) 125(21):2621–9. 10.1161/CIRCULATIONAHA.111.087940 22556340 PMC3365629

[14] Estes NAM. Computerized interpretation of ECGs. Circ Arrhythm Electrophysiol. (2013) 6(1):2–4. 10.1161/CIRCEP.111.000097 23424219

[15] Schläpfer J Wellens HJ. Computer-interpreted electrocardiograms. J Am Coll Cardiol. (2017) 70(9):1183–92. 10.1016/j.jacc.2017.07.723 28838369

[16] Shah AP Rubin SA. Errors in the computerized electrocardiogram interpretation of cardiac rhythm. J Electrocardiol. (2007) 40(5):385–90. 10.1016/j.jelectrocard.2007.03.008 17531257

[17] Willems JL Abreu-Lima C Arnaud P Van Bemmel JH Brohet C Degani R The diagnostic performance of computer programs for the interpretation of electrocardiograms. N Engl J Med. (1991) 325(25):1767–73. 10.1056/NEJM199112193252503 1834940

[18] Hongo RH Goldschlager N. Status of computerized electrocardiography. Cardiol Clin. (2006) 24(3):491–504. 10.1016/j.ccl.2006.03.005 16939838

[19] Mayourian J La Cava WG Vaid A Nadkarni GN Ghelani SJ Mannix R Pediatric ECG-based deep learning to predict left ventricular dysfunction and remodeling. Circulation. (2024) 149(12):917–31. 10.1161/CIRCULATIONAHA.123.067750 38314583 PMC10948312

[20] Chen J Huang S Zhang Y Chang Q Zhang Y Li D Congenital heart disease detection by pediatric electrocardiogram based deep learning integrated with human concepts. Nat Commun. (2024) 15(1):976. 10.1038/s41467-024-44930-y 38302502 PMC10834950

[21] Bos JM Attia ZI Albert DE Noseworthy PA Friedman PA Ackerman MJ. Use of artificial intelligence and deep neural networks in evaluation of patients with electrocardiographically concealed long QT syndrome from the surface 12-lead electrocardiogram. JAMA Cardiol. (2021) 6(5):532–8. 10.1001/jamacardio.2020.7422 33566059 PMC7876623

[22] Mori H Inai K Sugiyama H Muragaki Y. Diagnosing atrial septal defect from electrocardiogram with deep learning. Pediatr Cardiol. (2021) 42(6):1379–87. 10.1007/s00246-021-02622-0 33907875

[23] Campbell MJ Zhou X Han C Abrishami H Webster G Miyake CY Pilot study analyzing automated ECG screening of hypertrophic cardiomyopathy. Heart Rhythm. (2017) 14(6):848–52. 10.1016/j.hrthm.2017.02.011 28193509

[24] Prineas RJ Crow RS Zhang Z-M. The Minnesota Code Manual of Electrocardiographic Findings: Including Measurement and Comparison with the Novacode Standards and Procedures for ECG Measurement in Epidemiologic and Clinical Trials. 2nd ed. London: Springer (2010).

[25] Pan J Tompkins WJ. A real-time QRS detection algorithm. IEEE Trans Biomed Eng. (1985) 32(3):230–6. 10.1109/TBME.1985.325532 3997178

[26] Simonyan K Zisserman A. Very deep convolutional networks for large-scale image recognition. arXiv *[Preprint]*. (2024) arXiv:1409.1556. Available online at: 10.48550/arXiv.1409.1556 (Accessed July 28, 2024).

[27] Vickers AJ Elkin EB. Decision curve analysis: a novel method for evaluating prediction models. Med Decis Making. (2006) 26(6):565–74. 10.1177/0272989X06295361 17099194 PMC2577036

[28] Trajman A Luiz RR. Mcnemar *χ*^2^ test revisited: comparing sensitivity and specificity of diagnostic examinations. Scand J Clin Lab Invest. (2008) 68(1):77–80. 10.1080/00365510701666031 18224558

[29] Selvaraju RR Cogswell M Das A Vedantam R Parikh D Batra D. Grad-cam: visual explanations from deep networks via gradient-based localization. In: *Proceedings of the IEEE International Conference on Computer Vision 2017*; 2017 October 22–29; Venice, Italy. p. 618–26. 10.1109/ICCV.2017.74

[30] Schläpfer J Wellens HJ. Computer-interpreted electrocardiograms: benefits and limitations. J Am Coll Cardiol. (2017) 70(9):1183–92. 10.1016/j.jacc.2017.07.723 28838369

[31] Hong S Zhou Y Shang J Xiao C Sun J. Opportunities and challenges of deep learning methods for electrocardiogram data: a systematic review. Comput Biol Med. (2020) 122:103801. 10.1016/j.compbiomed.2020.103801 32658725

[32] Attia ZI Harmon DM Behr ER Friedman PA. Application of artificial intelligence to the electrocardiogram. Eur Heart J. (2021) 42(46):4717–30. 10.1093/eurheartj/ehab649 34534279 PMC8500024

[33] Siontis KC Noseworthy PA Attia ZI Friedman PA. Artificial intelligence-enhanced electrocardiography in cardiovascular disease management. Nat Rev Cardiol. (2021) 18(7):465–78. 10.1038/s41569-020-00503-2 33526938 PMC7848866

[34] Siontis KC Liu K Bos JM Attia ZI Cohen-Shelly M Arruda-Olson AM Detection of hypertrophic cardiomyopathy by an artificial intelligence electrocardiogram in children and adolescents. Int J Cardiol. (2021) 340:42–7. 10.1016/j.ijcard.2021.08.026 34419527

[35] Attia ZI Friedman PA Noseworthy PA Lopez-Jimenez F Ladewig DJ Satam G Age and sex estimation using artificial intelligence from standard 12-lead ECGs. Circ Arrhythm Electrophysiol. (2019) 12(9):e007284. 10.1161/CIRCEP.119.007284 31450977 PMC7661045

[36] Attia ZI Kapa S Lopez-Jimenez F McKie PM Ladewig DJ Satam G Screening for cardiac contractile dysfunction using an artificial intelligence–enabled electrocardiogram. Nat Med. (2019) 25(1):70–4. 10.1038/s41591-018-0240-2 30617318

[37] Attia ZI Noseworthy PA Lopez-Jimenez F Asirvatham SJ Deshmukh AJ Gersh BJ An artificial intelligence-enabled ECG algorithm for the identification of patients with atrial fibrillation during sinus rhythm: a retrospective analysis of outcome prediction. Lancet. (2019) 394(10201):861–7. 10.1016/S0140-6736(19)31721-0 31378392

[38] Goto S Kimura M Katsumata Y Goto S Kamatani T Ichihara G Artificial intelligence to predict needs for urgent revascularization from 12-leads electrocardiography in emergency patients. PLoS One. (2019) 14(1):e0210103. 10.1371/journal.pone.0210103 30625197 PMC6326503

[39] Baek Y-S Lee S-C Choi W Kim D-H. A new deep learning algorithm of 12-lead electrocardiogram for identifying atrial fibrillation during sinus rhythm. Sci Rep. (2021) 11(1):12818. 10.1038/s41598-021-92172-5 34140578 PMC8211689

[40] Huang J Chen B Yao B He W. ECG arrhythmia classification using STFT-based spectrogram and convolutional neural network. IEEE Access. (2019) 7:92871–80. 10.1109/ACCESS.2019.2928017

[41] Kagiyama N Piccirilli M Yanamala N Shrestha S Farjo PD Casaclang-Verzosa G Machine learning assessment of left ventricular diastolic function based on electrocardiographic features. J Am Coll Cardiol. (2020) 76(8):930–41. 10.1016/j.jacc.2020.06.061 32819467

[42] Cai W Chen Y Guo J Han B Shi Y Ji L Accurate detection of atrial fibrillation from 12-lead ECG using deep neural network. Comput Biol Med. (2020) 116:103378. 10.1016/j.compbiomed.2019.103378 31778896

[43] Ribeiro AH Ribeiro MH Paixão GMM Oliveira DM Gomes PR Canazart JA Automatic diagnosis of the 12-lead ECG using a deep neural network. Nat Commun. (2020) 11(1):1760. 10.1038/s41467-020-15432-4 32273514 PMC7145824

[44] Smith SW Walsh B Grauer K Wang K Rapin J Li J A deep neural network learning algorithm outperforms a conventional algorithm for emergency department electrocardiogram interpretation. J Electrocardiol. (2019) 52:88–95. 10.1016/j.jelectrocard.2018.11.013 30476648

